# Brain injury biomarkers and inflammatory markerslike pronostic factors on mortality in patients with spontaneous intracranial hemorrhage (medical-adults intensive care)

**DOI:** 10.1186/2197-425X-3-S1-A852

**Published:** 2015-10-01

**Authors:** DL Aguillón Prada, A Serrano Lázaro, RC Huerta Bravo, A Mesejo Arizmendi, JM Segura Roca, JC Sanchis Miñana, C Sanchis Piqueras, J Romero Guía, M Rodriguez Gimillo

**Affiliations:** Medical Intensive Care Unit, Hospital Clinic Universitario de Valencia, Valencia, Spain

## Objective

To value the usefulness of brain injury biomarkers (BIB) and inflammatory markers (IM) to predict mortality in spontaneous intracranial hemorrhage patients (SIH).

## Material and Methods

BIB (D Dimer (DD), BNP and CRP) were determined at admission, 1, 2, 3 and 7th day; IM at admission and 7th day. Descriptive analysis % and Median (minimal/ maximum). Independent samples T-student to compare means (p < 0.05). A binary logistic multivariate regression analysis was performed (95% CI OR).

## Results

103 patients with SIH. 66% were men. Age 61.8 (± 12.7). Overall Mortallity 37%. ICU stay 7 days (1-55). Glasgow Coma Scale 12 (3-15). SIH 20.18 cc volume (1-252).

The univariate correlation between of BIB and MI with mortality in Table 1. In mutivariate analysis: protective factors were BNP at admission (OR 1.1, 95% CI 1.1-1.2 (p 0.02)), DD at 7th day (OR 1.1, 95% CI 1.1-1.3 (p 0.009)) and Prealbumin at 7th day (OR 0.9, 95% CI 0.8-0.9 (p 0.03)).

**Table 1:** Relationship brain injury biomarkers and inflammatory markers with mortality; Median ± SD. BIB: Brain injury biomarkers; IM: Inflammatory markers; BNP: Brain natriuretic peptide; DD: D Dimer.

BIB and IM		Dead	Alive	P
BNP (pg/ml)	Admission	223,5 ± 318,46	82.41 ± 95,55	0,001
	24 h	175,96 ± 202,08	87.84 ± 77,19	0,002
	48 h	142.32 ± 156,01	94.12 ± 82,66	0,04
DD (µg/L)	48 h	1915,8 ± 1631,5	883,7 ± 1368,6	0, 001
	72 h	2525,27 ± 2142,47	1051.92 ± 1254,15	0,000
	7th d	3677.07 ± 2224,72	2150.9 ± 1820,42	0,005
Leukocytosis	Admission	12037,5 ± 4806,8	10241,8 ± 3624,2	0,03
Albumin (g/dl)	Admission	3,4 ± 0,52	3,7 ± 0,55	0,01
Prealbúmin (mg/dl)	Admission 7th d	18,97 ± 6,78 16,74 ± 7,28	22.30 ± 5,36 21,22 ± 8,03	0,008 0,02

## Conclusions

BNP at admission, 24 h and 48 h and DD as late marker at 48 h, 72 h and 7th day were correlated with mortality in patients with SIH. IM correlated with mortality: leukocytosis, albumin and prealbumin at admission and prealbumin, ferritin and haptoglobin at 7th day.

Multivariate analysis found significance for increased risk of death BNP at admission and DD at 7th day; and protective factor was Prealbumin at 7th day.

